# Ureteroscopy-Assisted Retrograde Nephrostomy (UARN) after Anatrophic Nephrolithotomy

**DOI:** 10.1155/2012/164963

**Published:** 2012-08-09

**Authors:** Takashi Kawahara, Hiroki Ito, Hideyuki Terao, Yoshitake Kato, Takehiko Ogawa, Hiroji Uemura, Yoshinobu Kubota, Junichi Matsuzaki

**Affiliations:** ^1^Department of Urology, Ohguchi Higashi General Hospital, 2-19-1 Irie, Kanagawa-ku, Yokohama, Kanagawa, Japan; ^2^Department of Urology, Yokohama City University Graduate School of Medicine, Yokohama, Japan

## Abstract

*Introduction*. Open surgical anatrophic nephrolithotomy (ANL) had been the standard treatment for large renal calculi prior to the development of endoscopic devices and endoscopic techniques. A previous report described the efficacy of ureteroscopy-assisted retrograde nephrostomy (UARN) and presented a case of renal calculi successfully treated with UARN during percutaneous nephrolithotomy (PCNL) in a patient after ANL. *Case Presentation*. A 61-year-old male with left renal calculi was referred for further treatment. The patient was placed under general and epidural anesthesia, in a Galdakao-modified Valdivia position. A flexible ureteroscope (URS) was inserted, and a Lawson retrograde nephrostomy puncture wire was advanced into the flexible URS. The puncture wire then followed the route from the renal pelvis to the exit skin. Calculus fragmentation was undertaken using a pneumatic lithotripter. *Conclusions*. UARN for PCNL was therefore found to be a safe, effective, and appropriate treatment for a patient presenting with renal calculi after undergoing ANL.

## 1. Introduction

 Staghorn calculi are branched and generally infected stones that occupy a large portion of the renal collecting system [1]. Management of staghorn calculus remains a challenge even using endoscopic devices and techniques [2]. Open surgical anatrophic nephrolithotomy (ANL) had been the standard treatment of large renal stones prior to the development of endoscopic procedures. The progressive use of laparoscopic techniques has led to the use of laparoscopic ANL [2–4].

 Goodwin et al. first reported percutaneous renal access in 1955 [5]. Percutaneous nephrolithotomy (PCNL) was developed, and PCNL became the standard procedure for large renal stones [6]. Ureteroscopy (URS)-assisted retrograde nephrostomy (UARN) has been described [7]. UARN allows the surgeon to continuously visualize the dilation from the initial puncture to the insertion of the nephroaccess sheath (NAS). This report presents a case of renal calculi successfully treated with UARN during PCNL in a patient that had previously undergone ANL.

## 2. Case Presentation

**Figure 1:**
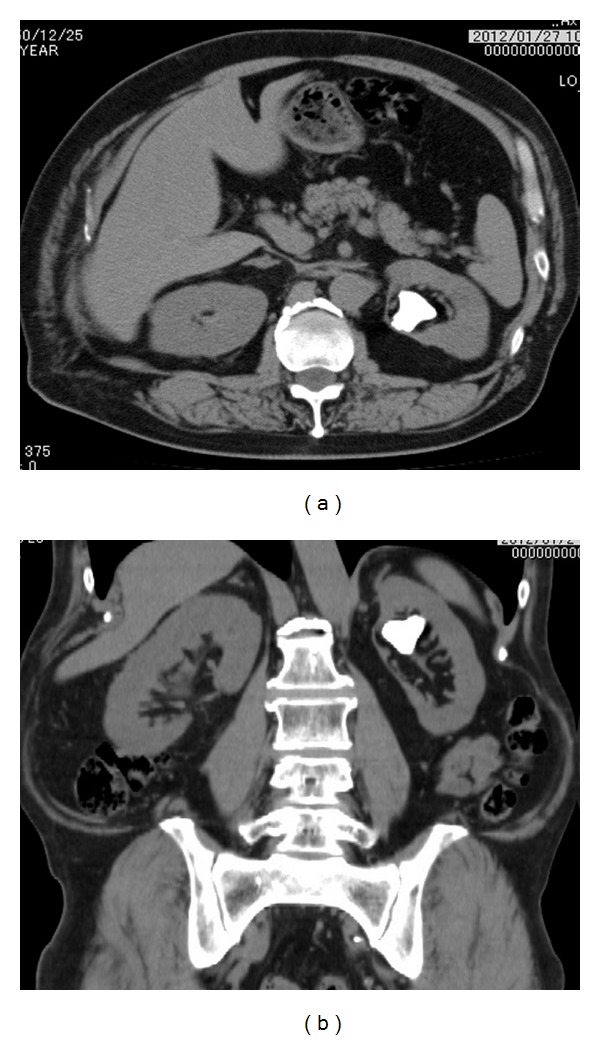
Preoperative axial (a) and coronal (b) noncontract CTKUB films.

 A 61-year-old male was referred in order to receive treatment of a left renal stone (Figure 1). He had undergone bilateral open surgical ANL 30 years earlier. His laboratory data showed no remarkable findings except for microhematuria on urinary analysis. The patient was admitted to for PCNL in April 2012 to treat his left renal calculus.

**Figure 2:**
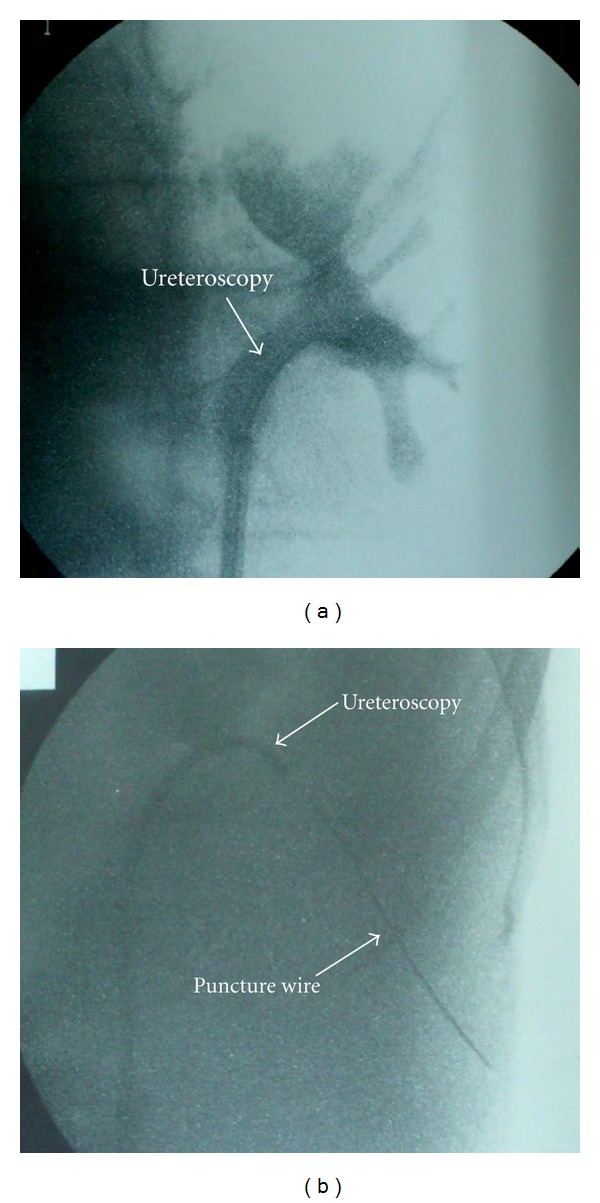
Retrograde pyelography (a) and advancing puncture wire to the skin (b).

**Figure 3:**
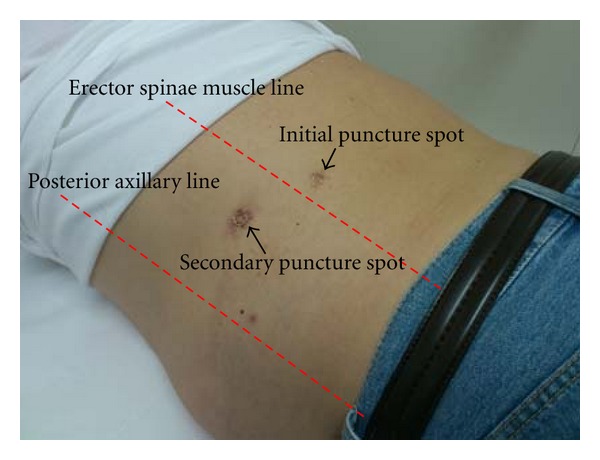
Initial puncture spot at skin is too dorsally.

PCNL was performed using the UARN technique. The technique was performed as described in previous reports [7, 8]. The patient was placed under general and epidural anesthesia, in a modified Valdivia position (Galdakao-modified Valdivia position) [9]. The ureteral access sheath (UAS; Navigator 11 Fr, 46 cm, Boston Scientific, Natick, MA, USA) was inserted into the ureter, and flexible ureteroscopy (URS; Flex-X^2^, Karl Storz, Tuttlingen, Germany) was carried out to observe the anatomical image between the target stone and renal collecting system. The target calyx to the skin was checked using ultrasonography before puncturing, to avoid puncturing the surrounding organs. The Lawson retrograde nephrostomy puncture wire (Cook Urological, Bloomington, IN, USA) was set with URS and advanced to the target calyx and advanced to the skin under fluoroscopic guidance (Figure 2). Most procedures have indicated that the middle calyx in the dorsal side is a suitable spot to puncture. This calyx was punctured; however, the “tent sign” at the skin surface was too dorsal and risked erector spinae muscle invasion. Therefore, the middle calyx on the ventral side was selected as the target calyx. The puncture wire passed through the renal capsule easily and then tented the skin at the posterior axillary line (Figure 3). A 24 Fr percutaneous nephro access sheath (NAS; X-Force Nephrostomy Balloon Dilation Catheter, BARD, Murray Hill, NJ, USA) was passed over the balloon under continuous visualization with the URS. The NAS was inserted into the renal collecting system, and calculus fragmentation was performed using the Swiss Litho Clast pneumatic lithotripter (EMS, Nyon, Switzerland) through a rigid nephroscope (percutaneous nephroscope, Karl Storz, Tuttlingen, Germany). A ureteral stent was inserted at the conclusion of PCNL and then was removed two weeks after the operation. Computed tomography confirmed that the patient was stone free. The stone was found to be composed of calcium phosphate.

## 3. Discussion

The surgical management of urinary calculi disease has evolved from an open surgical approach to various minimally invasive options including URS, SWL, and PCNL [2, 10]. The treatment of patients with staghorn calculus remains among the most complex and challenging problems in urology [3]. ANL, with formal plastic calligraphy and/or calycoplasty, was described by Smith and Boyce in 1968 [11]. Matlaga and Assimos reported a stone-free rate of 100% using open stone surgery for staghorn renal stones [12]. Kaouk et al. were the first to report their experience with laparoscopic ANL in a porcine model [13]. ANL is recognized to be more reliable than SWL and PCNL in terms of stone removal when treating a large staghorn calculus, with stone-free rates of 80–100% [14, 15]. Laparoscopic ANL is a promising alternative for patients who are candidates for open surgery, with an acceptable stone-free rate [4].

 Repeated ANL for recurrent renal stones would be difficult because of the invasiveness of ANL and postoperative adhesions. Goodwin et al. first reported percutaneous renal access in 1955 [5]. PCNL was developed, and PCNL became the standard procedure for large renal stones [6]. The renal position was repositioned to the dorsal side, due to the intraoperative separation and postoperative adhesion associated with ANL. Puncturing from the middle calyx on the dorsal side was too dorsal.

The most critical issue in PCNL placement is the selection of the puncture site, to minimize the risk of hemorrhage, which is the most common major complication. US-guided puncture of the collecting system with subsequent placement of the drainage tube under fluoroscopic guidance is the standard modality for PCNL; however, US-guided nephrostomy is difficult with anatomical anomalies of the abdomen or kidney. Major advances have made the observation of the renal pelvis easier, making it possible to perform a wide variety of intrarenal procedures using an URS [16]. Therefore, it is easier to approach the desired renal calyx using a flexible URS than was possible using previous fluoroscopic approaches [17, 18].

More than 50 patients have so far been treated with UARN, including patients with complete staghorn calculi, horseshoe kidney, obese patients, patient with incomplete double ureter, and patients with ileal conduit urinary diversion [7, 8, 19]. UARN allows for the continuous visualization of the dilation from puncture to the insertion of the nephroaccess sheath (NAS). The retrograde nephrostomy puncture usually requires a single movement, and since the needle passes from a posteriorly located calyx through the retroperitoneum, the possibility of damage to intra- and extrarenal vessels is less likely [20]. A potential disadvantage of this procedure is the danger of exiting the kidney in the ventral direction, and thereby possibly injuring the intestines [20]. The puncture was made under ultrasonographic and fluoroscopic guidance to avoid injuring the surrounding organs. UARN is also a safe and effective approach for patients presenting with calculi after having previously undergone ANL.

## 4. Conclusions

UARN was an acceptable treatment for PCNL in a patient after ANL.
